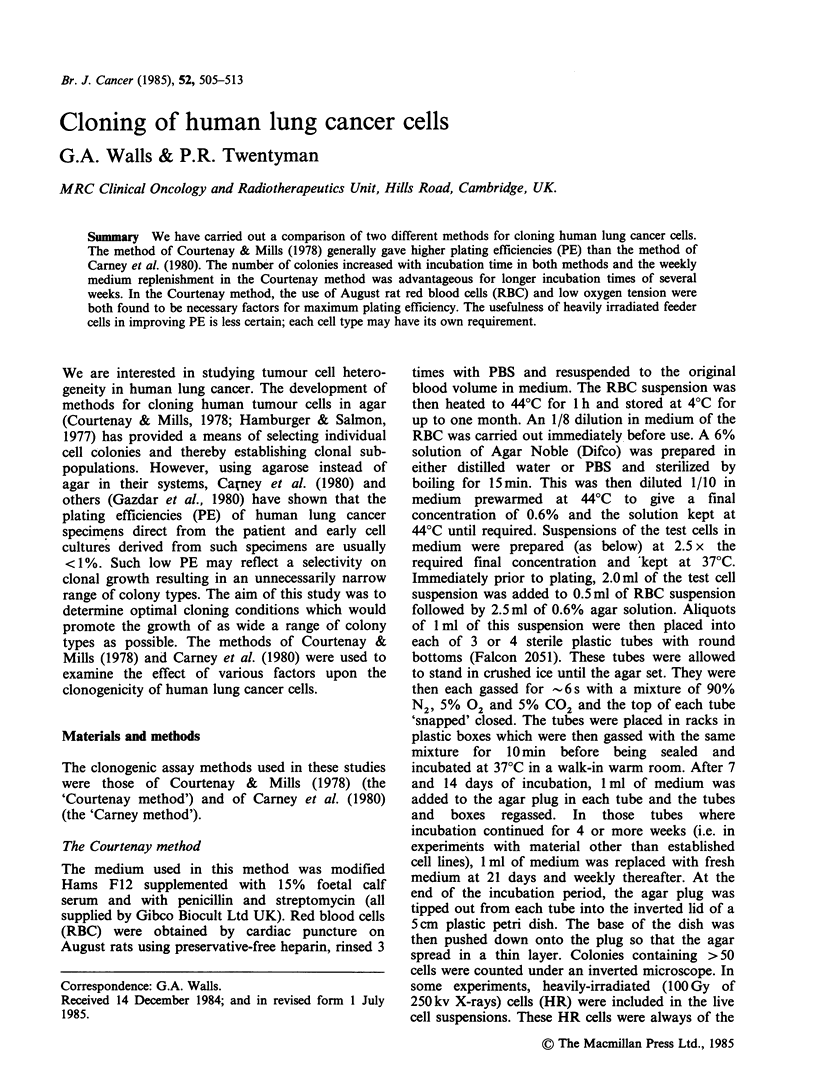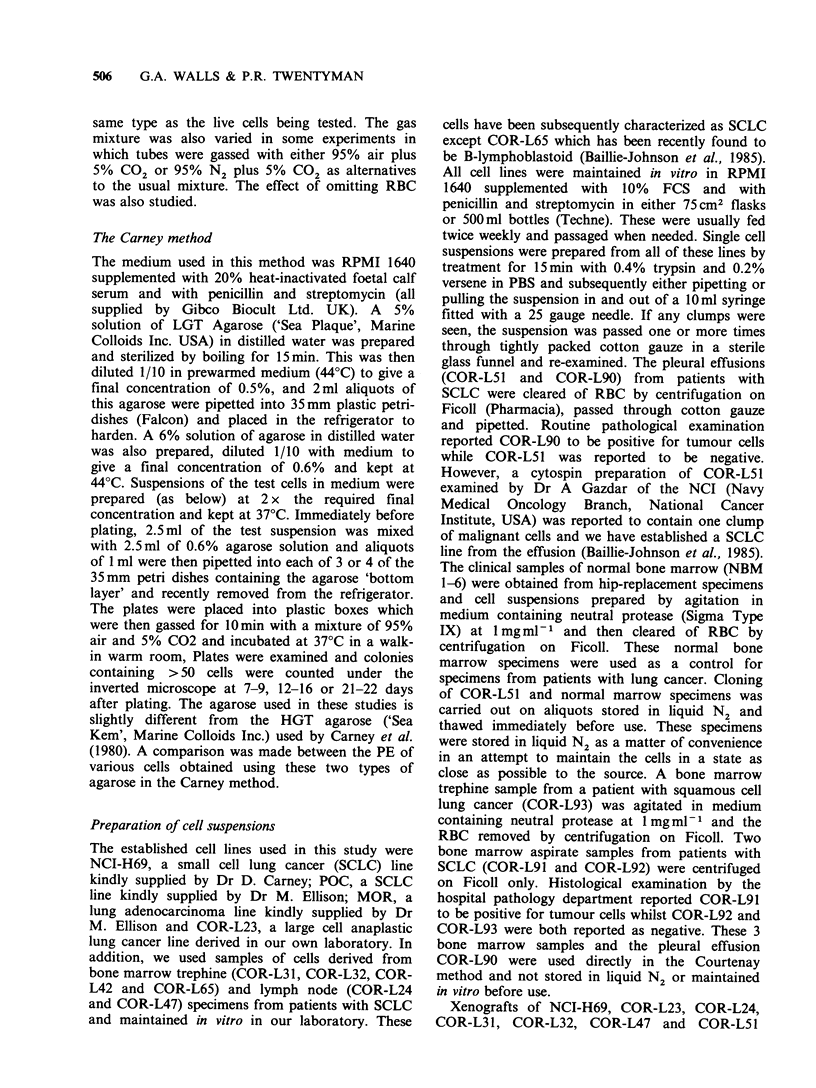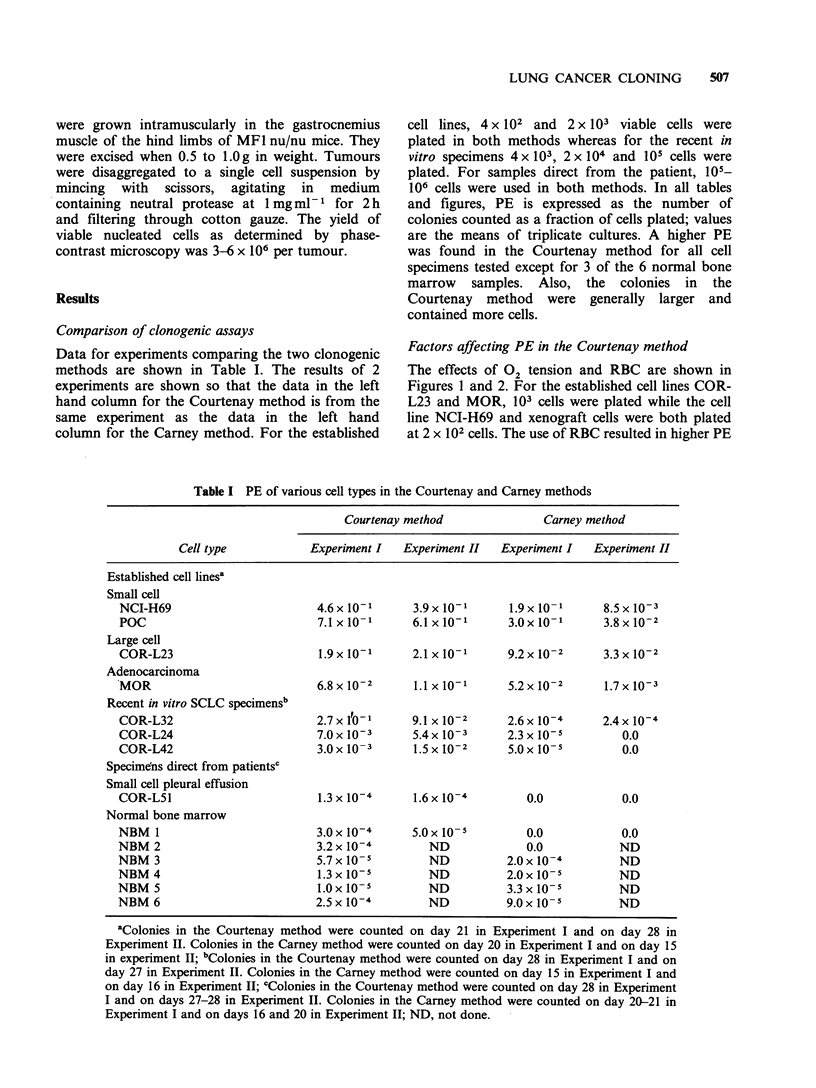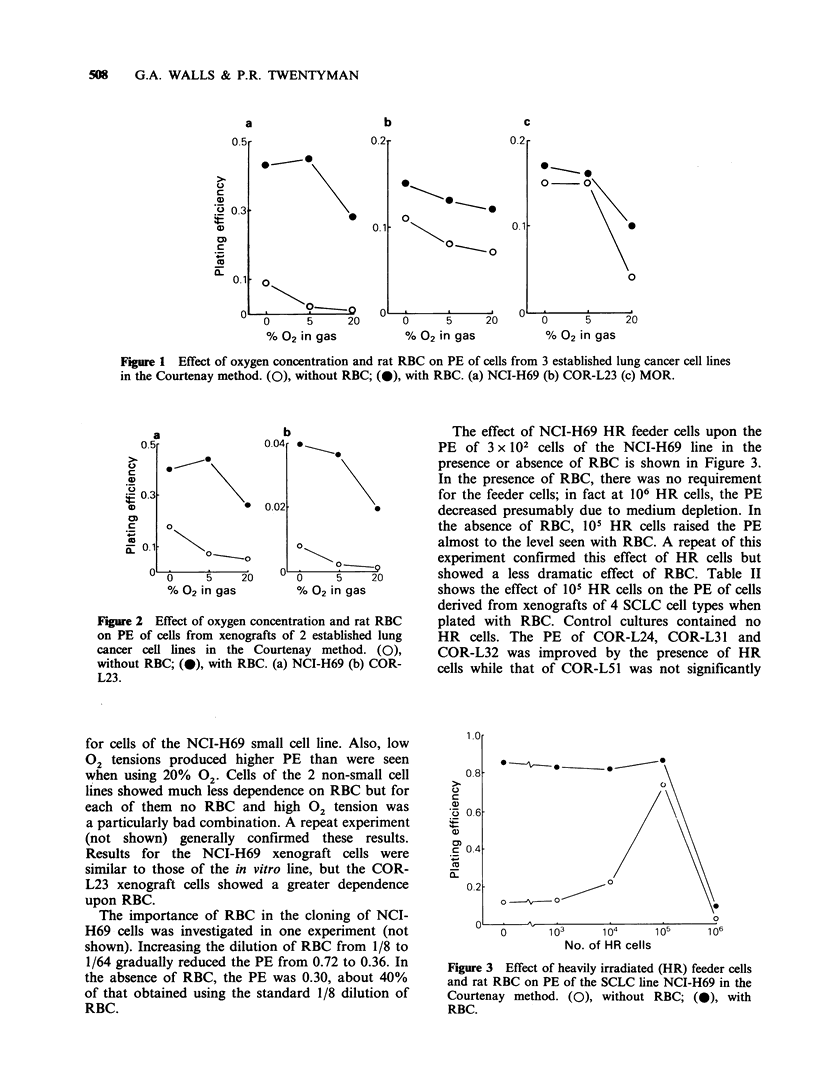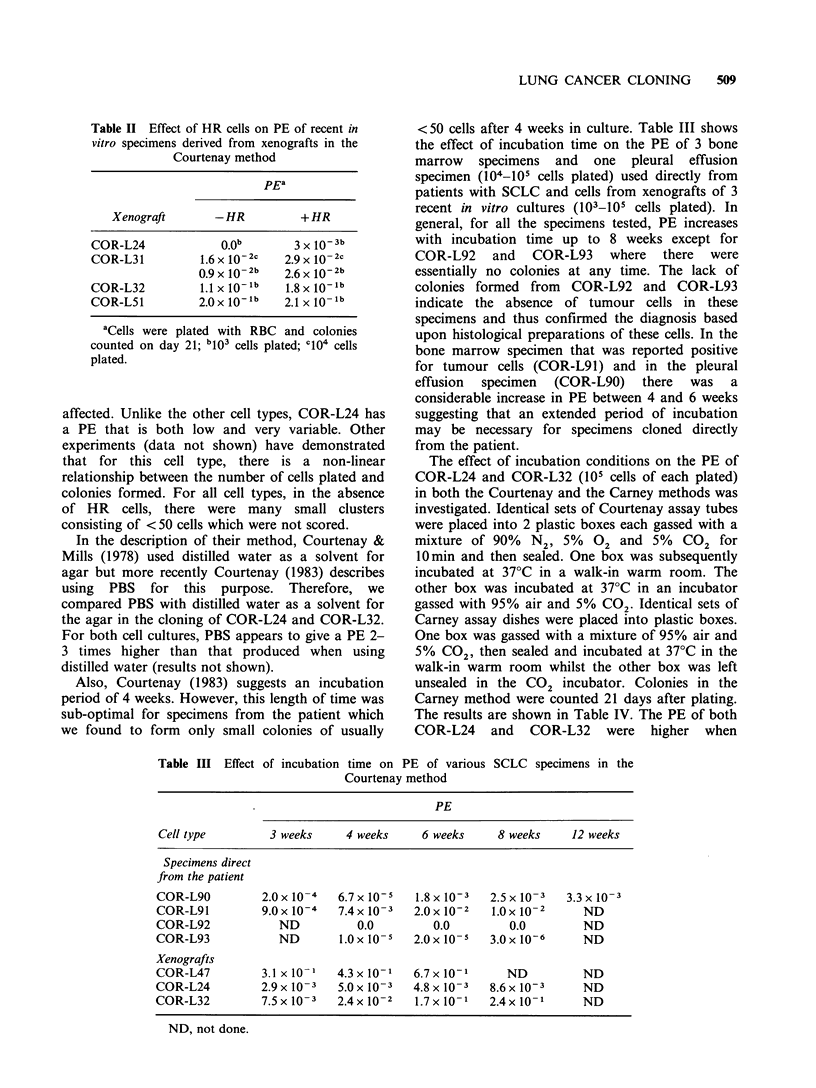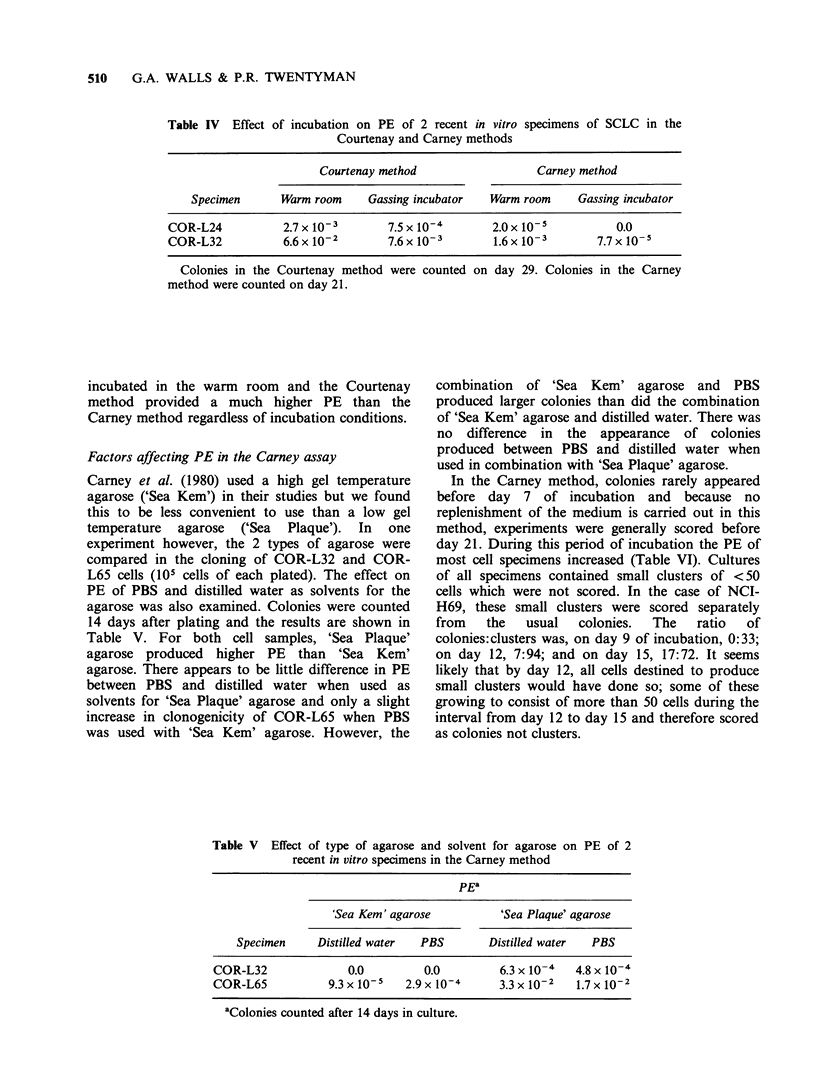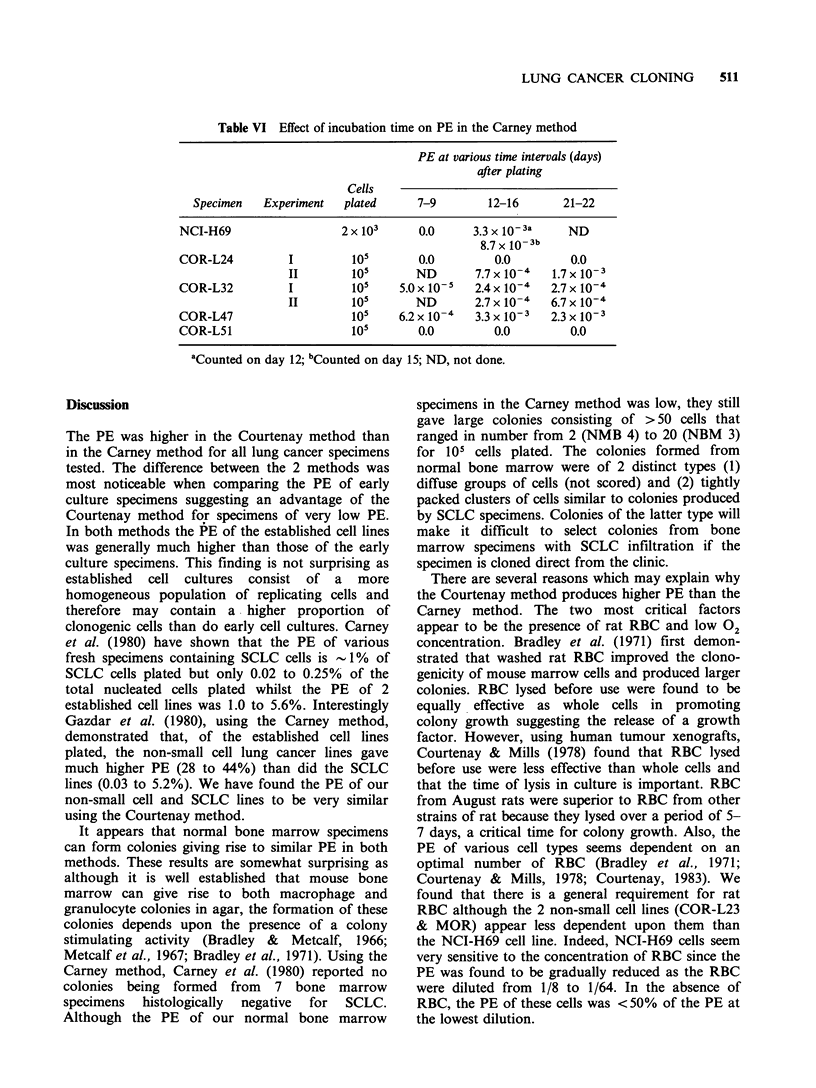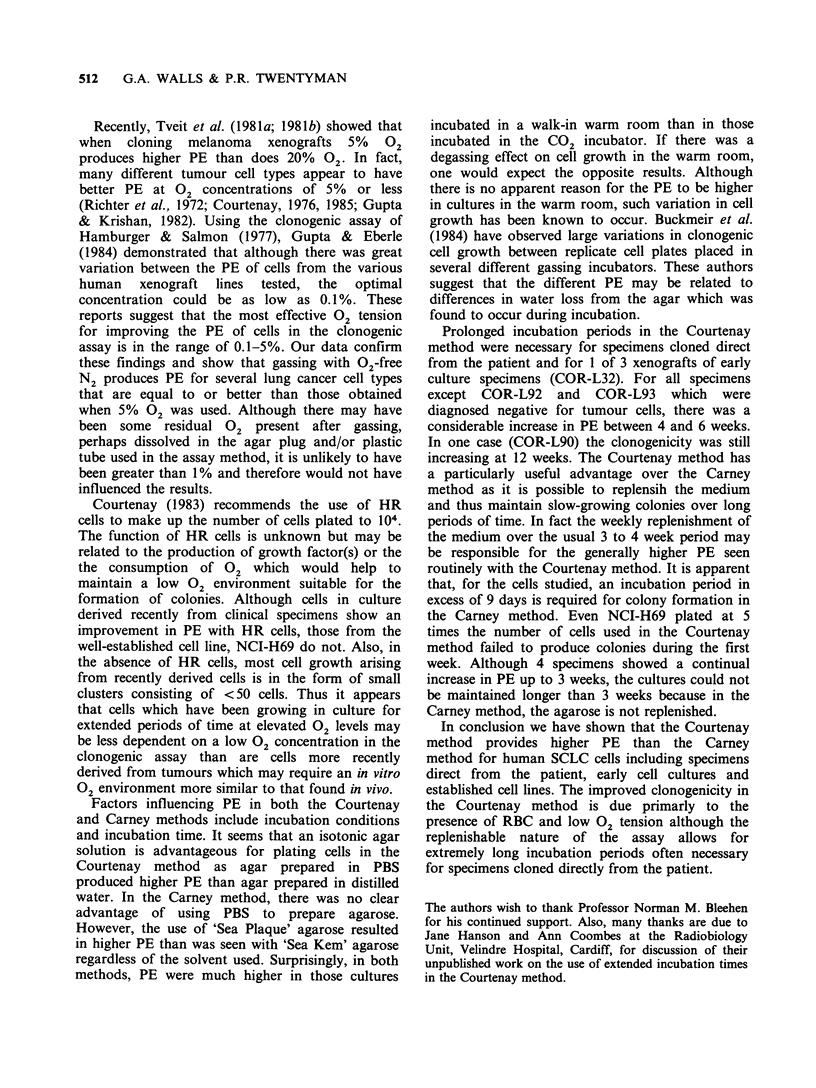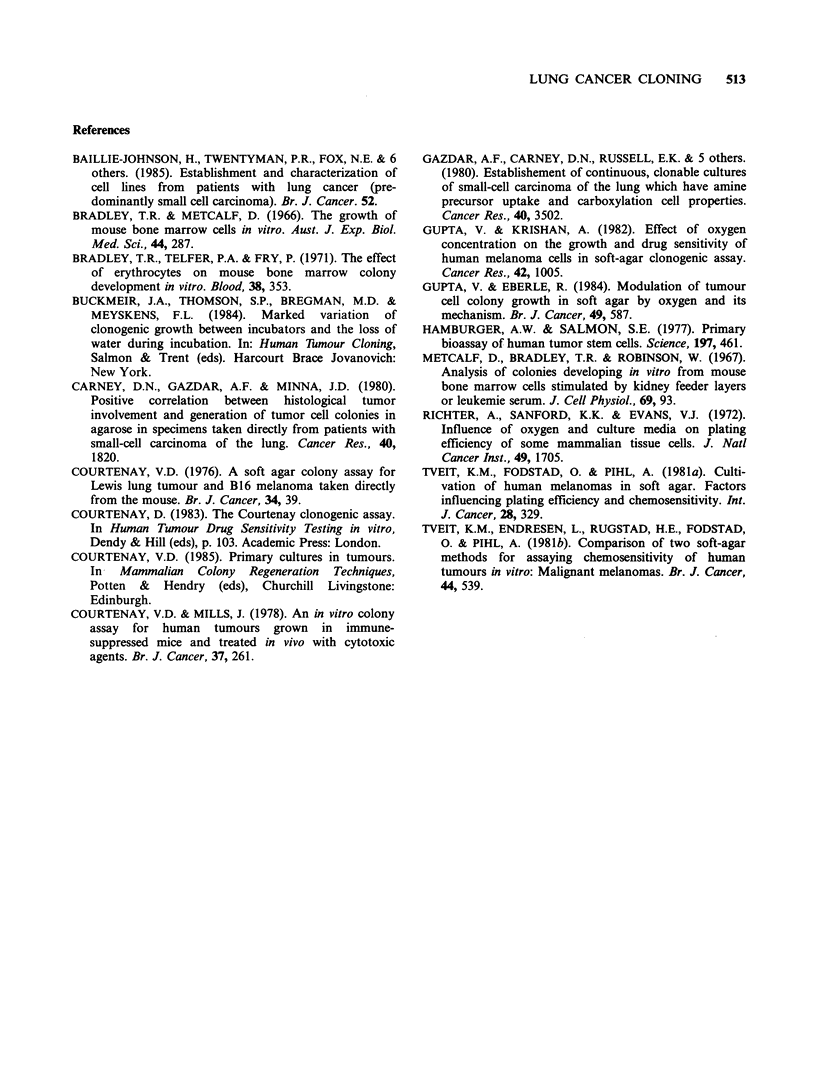# Cloning of human lung cancer cells.

**DOI:** 10.1038/bjc.1985.221

**Published:** 1985-10

**Authors:** G. A. Walls, P. R. Twentyman

## Abstract

We have carried out a comparison of two different methods for cloning human lung cancer cells. The method of Courtenay & Mills (1978) generally gave higher plating efficiencies (PE) than the method of Carney et al. (1980). The number of colonies increased with incubation time in both methods and the weekly medium replenishment in the Courtenay method was advantageous for longer incubation times of several weeks. In the Courtenay method, the use of August rat red blood cells (RBC) and low oxygen tension were both found to be necessary factors for maximum plating efficiency. The usefulness of heavily irradiated feeder cells in improving PE is less certain; each cell type may have its own requirement.


*Br. J. Cancer* (1985), **52**, 505–513

### Cloning of human lung cancer cells

G.A. Walls & P.R. Twentyman

*MRC Clinical Oncology and Radiotherapeutics Unit, Hills Road, Cambridge, UK.*

**Summary** We have carried out a comparison of two different methods for cloning human lung cancer cells. The method of Courtenay & Mills (1978) generally gave higher plating efficiencies (PE) than the method of Carney *et al.* (1980). The number of colonies increased with incubation time in both methods and the weekly medium replenishment in the Courtenay method was advantageous for longer incubation times of several weeks. In the Courtenay method, the use of August rat red blood cells (RBC) and low oxygen tension were both found to be necessary factors for maximum plating efficiency. The usefulness of heavily irradiated feeder cells in improving PE is less certain; each cell type may have its own requirement.

We are interested in studying tumour cell heterogeneity in human lung cancer. The development of methods for cloning human tumour cells in agar (Courtenay & Mills, 1978; Hamburger & Salmon, 1977) has provided a means of selecting individual cell colonies and thereby establishing clonal subpopulations. However, using agarose instead of agar in their systems, Carney *et al.* (1980) and others (Gazdar *et al.*, 1980) have shown that the plating efficiencies (PE) of human lung cancer specimens direct from the patient and early cell cultures derived from such specimens are usually <1%. Such low PE may reflect a selectivity on clonal growth resulting in an unnecessarily narrow range of colony types. The aim of this study was to determine optimal cloning conditions which would promote the growth of as wide a range of colony types as possible. The methods of Courtenay & Mills (1978) and Carney *et al.* (1980) were used to examine the effect of various factors upon the clonogenicity of human lung cancer cells.

### Materials and methods

The clonogenic assay methods used in these studies were those of Courtenay & Mills (1978) (the 'Courtenay method') and of Carney *et al.* (1980) (the 'Carney method').

### *The Courtenay method*

The medium used in this method was modified Hams F12 supplemented with 15% foetal calf serum and with penicillin and streptomycin (all supplied by Gibco Biocult Ltd UK). Red blood cells (RBC) were obtained by cardiac puncture on August rats using preservative-free heparin, rinsed 3 times with PBS and resuspended to the original blood volume in medium. The RBC suspension was then heated to 44°C for 1 h and stored at 4°C for up to one month. An 1/8 dilution in medium of the RBC was carried out immediately before use. A 6% solution of Agar Noble (Difco) was prepared in either distilled water or PBS and sterilized by boiling for 15 min. This was then diluted 1/10 in medium prewarmed at 44°C to give a final concentration of 0.6% and the solution kept at 44°C until required. Suspensions of the test cells in medium were prepared (as below) at 2.5× the required final concentration and kept at 37°C. Immediately prior to plating, 2.0 ml of the test cell suspension was added to 0.5 ml of RBC suspension followed by 2.5 ml of 0.6% agar solution. Aliquots of 1 ml of this suspension were then placed into each of 3 or 4 sterile plastic tubes with round bottoms (Falcon 2051). These tubes were allowed to stand in crushed ice until the agar set. They were then each gassed for ~6 s with a mixture of 90% $N_2$, 5% $O_2$ and 5% $CO_2$ and the top of each tube 'snapped' closed. The tubes were placed in racks in plastic boxes which were then gassed with the same mixture for 10 min before being sealed and incubated at 37°C in a walk-in warm room. After 7 and 14 days of incubation, 1 ml of medium was added to the agar plug in each tube and the tubes and boxes regassed. In those tubes where incubation continued for 4 or more weeks (i.e. in experiments with material other than established cell lines), 1 ml of medium was replaced with fresh medium at 21 days and weekly thereafter. At the end of the incubation period, the agar plug was tipped out from each tube into the inverted lid of a 5 cm plastic petri dish. The base of the dish was then pushed down onto the plug so that the agar spread in a thin layer. Colonies containing >50 cells were counted under an inverted microscope. In some experiments, heavily-irradiated (100 Gy of 250 kv X-rays) cells (HR) were included in the live cell suspensions. These HR cells were always of the

Correspondence: G.A. Walls.
Received 14 December 1984; and in revised form 1 July 1985.

© The Macmillan Press Ltd., 1985

same type as the live cells being tested. The gas mixture was also varied in some experiments in which tubes were gassed with either 95% air plus 5% $CO_2$ or 95% $N_2$ plus 5% $CO_2$ as alternatives to the usual mixture. The effect of omitting RBC was also studied.

*The Carney method*

The medium used in this method was RPMI 1640 supplemented with 20% heat-inactivated foetal calf serum and with penicillin and streptomycin (all supplied by Gibco Biocult Ltd. UK). A 5% solution of LGT Agarose ('Sea Plaque', Marine Colloids Inc. USA) in distilled water was prepared and sterilized by boiling for 15 min. This was then diluted 1/10 in prewarmed medium (44°C) to give a final concentration of 0.5%, and 2 ml aliquots of this agarose were pipetted into 35 mm plastic petri-dishes (Falcon) and placed in the refrigerator to harden. A 6% solution of agarose in distilled water was also prepared, diluted 1/10 with medium to give a final concentration of 0.6% and kept at 44°C. Suspensions of the test cells in medium were prepared (as below) at 2× the required final concentration and kept at 37°C. Immediately before plating, 2.5 ml of the test suspension was mixed with 2.5 ml of 0.6% agarose solution and aliquots of 1 ml were then pipetted into each of 3 or 4 of the 35 mm petri dishes containing the agarose 'bottom layer' and recently removed from the refrigerator. The plates were placed into plastic boxes which were then gassed for 10 min with a mixture of 95% air and 5% CO2 and incubated at 37°C in a walk-in warm room, Plates were examined and colonies containing >50 cells were counted under the inverted microscope at 7–9, 12–16 or 21–22 days after plating. The agarose used in these studies is slightly different from the HGT agarose ('Sea Kem', Marine Colloids Inc.) used by Carney *et al.* (1980). A comparison was made between the PE of various cells obtained using these two types of agarose in the Carney method.

*Preparation of cell suspensions*

The established cell lines used in this study were NCI-H69, a small cell lung cancer (SCLC) line kindly supplied by Dr D. Carney; POC, a SCLC line kindly supplied by Dr M. Ellison; MOR, a lung adenocarcinoma line kindly supplied by Dr M. Ellison and COR-L23, a large cell anaplastic lung cancer line derived in our own laboratory. In addition, we used samples of cells derived from bone marrow trephine (COR-L31, COR-L32, COR-L42 and COR-L65) and lymph node (COR-L24 and COR-L47) specimens from patients with SCLC and maintained *in vitro* in our laboratory. These cells have been subsequently characterized as SCLC except COR-L65 which has been recently found to be B-lymphoblastoid (Baillie-Johnson *et al.*, 1985). All cell lines were maintained *in vitro* in RPMI 1640 supplemented with 10% FCS and with penicillin and streptomycin in either 75 $cm^2$ flasks or 500 ml bottles (Techne). These were usually fed twice weekly and passaged when needed. Single cell suspensions were prepared from all of these lines by treatment for 15 min with 0.4% trypsin and 0.2% versene in PBS and subsequently either pipetting or pulling the suspension in and out of a 10 ml syringe fitted with a 25 gauge needle. If any clumps were seen, the suspension was passed one or more times through tightly packed cotton gauze in a sterile glass funnel and re-examined. The pleural effusions (COR-L51 and COR-L90) from patients with SCLC were cleared of RBC by centrifugation on Ficoll (Pharmacia), passed through cotton gauze and pipetted. Routine pathological examination reported COR-L90 to be positive for tumour cells while COR-L51 was reported to be negative. However, a cytospin preparation of COR-L51 examined by Dr A Gazdar of the NCI (Navy Medical Oncology Branch, National Cancer Institute, USA) was reported to contain one clump of malignant cells and we have established a SCLC line from the effusion (Baillie-Johnson *et al.*, 1985). The clinical samples of normal bone marrow (NBM 1–6) were obtained from hip-replacement specimens and cell suspensions prepared by agitation in medium containing neutral protease (Sigma Type IX) at 1 mg $ml^{-1}$ and then cleared of RBC by centrifugation on Ficoll. These normal bone marrow specimens were used as a control for specimens from patients with lung cancer. Cloning of COR-L51 and normal marrow specimens was carried out on aliquots stored in liquid $N_2$ and thawed immediately before use. These specimens were stored in liquid $N_2$ as a matter of convenience in an attempt to maintain the cells in a state as close as possible to the source. A bone marrow trephine sample from a patient with squamous cell lung cancer (COR-L93) was agitated in medium containing neutral protease at 1 mg $ml^{-1}$ and the RBC removed by centrifugation on Ficoll. Two bone marrow aspirate samples from patients with SCLC (COR-L91 and COR-L92) were centrifuged on Ficoll only. Histological examination by the hospital pathology department reported COR-L91 to be positive for tumour cells whilst COR-L92 and COR-L93 were both reported as negative. These 3 bone marrow samples and the pleural effusion COR-L90 were used directly in the Courtenay method and not stored in liquid $N_2$ or maintained *in vitro* before use.

Xenografts of NCI-H69, COR-L23, COR-L24, COR-L31, COR-L32, COR-L47 and COR-L51

were grown intramuscularly in the gastrocnemius muscle of the hind limbs of MF1 nu/nu mice. They were excised when 0.5 to 1.0 g in weight. Tumours were disaggregated to a single cell suspension by mincing with scissors, agitating in medium containing neutral protease at $1 \text{ mg ml}^{-1}$ for 2 h and filtering through cotton gauze. The yield of viable nucleated cells as determined by phase-contrast microscopy was $3–6 \times 10^6$ per tumour.

### Results

### *Comparison of clonogenic assays*

Data for experiments comparing the two clonogenic methods are shown in Table I. The results of 2 experiments are shown so that the data in the left hand column for the Courtenay method is from the same experiment as the data in the left hand column for the Carney method. For the established cell lines, $4 \times 10^2$ and $2 \times 10^3$ viable cells were plated in both methods whereas for the recent *in vitro* specimens $4 \times 10^3$, $2 \times 10^4$ and $10^5$ cells were plated. For samples direct from the patient, $10^5$–$10^6$ cells were used in both methods. In all tables and figures, PE is expressed as the number of colonies counted as a fraction of cells plated; values are the means of triplicate cultures. A higher PE was found in the Courtenay method for all cell specimens tested except for 3 of the 6 normal bone marrow samples. Also, the colonies in the Courtenay method were generally larger and contained more cells.

### *Factors affecting PE in the Courtenay method*

The effects of $O_2$ tension and RBC are shown in Figures 1 and 2. For the established cell lines COR-L23 and MOR, $10^3$ cells were plated while the cell line NCI-H69 and xenograft cells were both plated at $2 \times 10^2$ cells. The use of RBC resulted in higher PE

**Table I** PE of various cell types in the Courtenay and Carney methods

| | *Courtenay method* | | *Carney method* | |
|---|---|---|---|---|
| *Cell type* | *Experiment I* | *Experiment II* | *Experiment I* | *Experiment II* |
| Established cell lines[a] | | | | |
| Small cell | | | | |
| NCI-H69 | $4.6 \times 10^{-1}$ | $3.9 \times 10^{-1}$ | $1.9 \times 10^{-1}$ | $8.5 \times 10^{-3}$ |
| POC | $7.1 \times 10^{-1}$ | $6.1 \times 10^{-1}$ | $3.0 \times 10^{-1}$ | $3.8 \times 10^{-2}$ |
| Large cell | | | | |
| COR-L23 | $1.9 \times 10^{-1}$ | $2.1 \times 10^{-1}$ | $9.2 \times 10^{-2}$ | $3.3 \times 10^{-2}$ |
| Adenocarcinoma | | | | |
| MOR | $6.8 \times 10^{-2}$ | $1.1 \times 10^{-1}$ | $5.2 \times 10^{-2}$ | $1.7 \times 10^{-3}$ |
| Recent *in vitro* SCLC specimens[b] | | | | |
| COR-L32 | $2.7 \times 10^{-1}$ | $9.1 \times 10^{-2}$ | $2.6 \times 10^{-4}$ | $2.4 \times 10^{-4}$ |
| COR-L24 | $7.0 \times 10^{-3}$ | $5.4 \times 10^{-3}$ | $2.3 \times 10^{-5}$ | 0.0 |
| COR-L42 | $3.0 \times 10^{-3}$ | $1.5 \times 10^{-2}$ | $5.0 \times 10^{-5}$ | 0.0 |
| Specimens direct from patients[c] | | | | |
| Small cell pleural effusion | | | | |
| COR-L51 | $1.3 \times 10^{-4}$ | $1.6 \times 10^{-4}$ | 0.0 | 0.0 |
| Normal bone marrow | | | | |
| NBM 1 | $3.0 \times 10^{-4}$ | $5.0 \times 10^{-5}$ | 0.0 | 0.0 |
| NBM 2 | $3.2 \times 10^{-4}$ | ND | 0.0 | ND |
| NBM 3 | $5.7 \times 10^{-5}$ | ND | $2.0 \times 10^{-4}$ | ND |
| NBM 4 | $1.3 \times 10^{-5}$ | ND | $2.0 \times 10^{-5}$ | ND |
| NBM 5 | $1.0 \times 10^{-5}$ | ND | $3.3 \times 10^{-5}$ | ND |
| NBM 6 | $2.5 \times 10^{-4}$ | ND | $9.0 \times 10^{-5}$ | ND |

[a]Colonies in the Courtenay method were counted on day 21 in Experiment I and on day 28 in Experiment II. Colonies in the Carney method were counted on day 20 in Experiment I and on day 15 in experiment II; [b]Colonies in the Courtenay method were counted on day 28 in Experiment I and on day 27 in Experiment II. Colonies in the Carney method were counted on day 15 in Experiment I and on day 16 in Experiment II; [c]Colonies in the Courtenay method were counted on day 28 in Experiment I and on days 27–28 in Experiment II. Colonies in the Carney method were counted on day 20–21 in Experiment I and on days 16 and 20 in Experiment II; ND, not done.

**Figure 1** Effect of oxygen concentration and rat RBC on PE of cells from 3 established lung cancer cell lines in the Courtenay method. (○), without RBC; (●), with RBC. (a) NCI-H69 (b) COR-L23 (c) MOR.

**Figure 2** Effect of oxygen concentration and rat RBC on PE of cells from xenografts of 2 established lung cancer cell lines in the Courtenay method. (○), without RBC; (●), with RBC. (a) NCI-H69 (b) COR-L23.

for cells of the NCI-H69 small cell line. Also, low $O_2$ tensions produced higher PE than were seen when using 20% $O_2$. Cells of the 2 non-small cell lines showed much less dependence on RBC but for each of them no RBC and high $O_2$ tension was a particularly bad combination. A repeat experiment (not shown) generally confirmed these results. Results for the NCI-H69 xenograft cells were similar to those of the *in vitro* line, but the COR-L23 xenograft cells showed a greater dependence upon RBC.

The importance of RBC in the cloning of NCI-H69 cells was investigated in one experiment (not shown). Increasing the dilution of RBC from 1/8 to 1/64 gradually reduced the PE from 0.72 to 0.36. In the absence of RBC, the PE was 0.30, about 40% of that obtained using the standard 1/8 dilution of RBC.

The effect of NCI-H69 HR feeder cells upon the PE of $3 \times 10^2$ cells of the NCI-H69 line in the presence or absence of RBC is shown in Figure 3. In the presence of RBC, there was no requirement for the feeder cells; in fact at $10^6$ HR cells, the PE decreased presumably due to medium depletion. In the absence of RBC, $10^5$ HR cells raised the PE almost to the level seen with RBC. A repeat of this experiment confirmed this effect of HR cells but showed a less dramatic effect of RBC. Table II shows the effect of $10^5$ HR cells on the PE of cells derived from xenografts of 4 SCLC cell types when plated with RBC. Control cultures contained no HR cells. The PE of COR-L24, COR-L31 and COR-L32 was improved by the presence of HR cells while that of COR-L51 was not significantly

**Figure 3** Effect of heavily irradiated (HR) feeder cells and rat RBC on PE of the SCLC line NCI-H69 in the Courtenay method. (○), without RBC; (●), with RBC.

**Table II** Effect of HR cells on PE of recent *in vitro* specimens derived from xenografts in the Courtenay method

| | $PE^{a}$ | |
|---|---|---|
| *Xenograft* | *−HR* | *+HR* |
| COR-L24 | $0.0^{b}$ | $3\times10^{-3b}$ |
| COR-L31 | $1.6\times10^{-2c}$ | $2.9\times10^{-2c}$ |
| | $0.9\times10^{-2b}$ | $2.6\times10^{-2b}$ |
| COR-L32 | $1.1\times10^{-1b}$ | $1.8\times10^{-1b}$ |
| COR-L51 | $2.0\times10^{-1b}$ | $2.1\times10^{-1b}$ |

[a]Cells were plated with RBC and colonies counted on day 21; [b]$10^3$ cells plated; [c]$10^4$ cells plated.

affected. Unlike the other cell types, COR-L24 has a PE that is both low and very variable. Other experiments (data not shown) have demonstrated that for this cell type, there is a non-linear relationship between the number of cells plated and colonies formed. For all cell types, in the absence of HR cells, there were many small clusters consisting of <50 cells which were not scored.

In the description of their method, Courtenay & Mills (1978) used distilled water as a solvent for agar but more recently Courtenay (1983) describes using PBS for this purpose. Therefore, we compared PBS with distilled water as a solvent for the agar in the cloning of COR-L24 and COR-L32. For both cell cultures, PBS appears to give a PE 2–3 times higher than that produced when using distilled water (results not shown).

Also, Courtenay (1983) suggests an incubation period of 4 weeks. However, this length of time was sub-optimal for specimens from the patient which we found to form only small colonies of usually <50 cells after 4 weeks in culture. Table III shows the effect of incubation time on the PE of 3 bone marrow specimens and one pleural effusion specimen ($10^4$–$10^5$ cells plated) used directly from patients with SCLC and cells from xenografts of 3 recent *in vitro* cultures ($10^3$–$10^5$ cells plated). In general, for all the specimens tested, PE increases with incubation time up to 8 weeks except for COR-L92 and COR-L93 where there were essentially no colonies at any time. The lack of colonies formed from COR-L92 and COR-L93 indicate the absence of tumour cells in these specimens and thus confirmed the diagnosis based upon histological preparations of these cells. In the bone marrow specimen that was reported positive for tumour cells (COR-L91) and in the pleural effusion specimen (COR-L90) there was a considerable increase in PE between 4 and 6 weeks suggesting that an extended period of incubation may be necessary for specimens cloned directly from the patient.

The effect of incubation conditions on the PE of COR-L24 and COR-L32 ($10^5$ cells of each plated) in both the Courtenay and the Carney methods was investigated. Identical sets of Courtenay assay tubes were placed into 2 plastic boxes each gassed with a mixture of 90% $N_2$, 5% $O_2$ and 5% $CO_2$ for 10 min and then sealed. One box was subsequently incubated at 37°C in a walk-in warm room. The other box was incubated at 37°C in an incubator gassed with 95% air and 5% $CO_2$. Identical sets of Carney assay dishes were placed into plastic boxes. One box was gassed with a mixture of 95% air and 5% $CO_2$, then sealed and incubated at 37°C in the walk-in warm room whilst the other box was left unsealed in the $CO_2$ incubator. Colonies in the Carney method were counted 21 days after plating. The results are shown in Table IV. The PE of both COR-L24 and COR-L32 were higher when

**Table III** Effect of incubation time on PE of various SCLC specimens in the Courtenay method

| | *PE* | | | | |
|---|---|---|---|---|---|
| *Cell type* | *3 weeks* | *4 weeks* | *6 weeks* | *8 weeks* | *12 weeks* |
| *Specimens direct from the patient* | | | | | |
| COR-L90 | $2.0\times10^{-4}$ | $6.7\times10^{-5}$ | $1.8\times10^{-3}$ | $2.5\times10^{-3}$ | $3.3\times10^{-3}$ |
| COR-L91 | $9.0\times10^{-4}$ | $7.4\times10^{-3}$ | $2.0\times10^{-2}$ | $1.0\times10^{-2}$ | ND |
| COR-L92 | ND | 0.0 | 0.0 | 0.0 | ND |
| COR-L93 | ND | $1.0\times10^{-5}$ | $2.0\times10^{-5}$ | $3.0\times10^{-6}$ | ND |
| *Xenografts* | | | | | |
| COR-L47 | $3.1\times10^{-1}$ | $4.3\times10^{-1}$ | $6.7\times10^{-1}$ | ND | ND |
| COR-L24 | $2.9\times10^{-3}$ | $5.0\times10^{-3}$ | $4.8\times10^{-3}$ | $8.6\times10^{-3}$ | ND |
| COR-L32 | $7.5\times10^{-3}$ | $2.4\times10^{-2}$ | $1.7\times10^{-1}$ | $2.4\times10^{-1}$ | ND |

ND, not done.

**Table IV** Effect of incubation on PE of 2 recent *in vitro* specimens of SCLC in the Courtenay and Carney methods

| | *Courtenay method* | | *Carney method* | |
|---|---|---|---|---|
| *Specimen* | *Warm room* | *Gassing incubator* | *Warm room* | *Gassing incubator* |
| COR-L24 | $2.7 \times 10^{-3}$ | $7.5 \times 10^{-4}$ | $2.0 \times 10^{-5}$ | 0.0 |
| COR-L32 | $6.6 \times 10^{-2}$ | $7.6 \times 10^{-3}$ | $1.6 \times 10^{-3}$ | $7.7 \times 10^{-5}$ |

Colonies in the Courtenay method were counted on day 29. Colonies in the Carney method were counted on day 21.

incubated in the warm room and the Courtenay method provided a much higher PE than the Carney method regardless of incubation conditions.

### *Factors affecting PE in the Carney assay*

Carney *et al.* (1980) used a high gel temperature agarose ('Sea Kem') in their studies but we found this to be less convenient to use than a low gel temperature agarose ('Sea Plaque'). In one experiment however, the 2 types of agarose were compared in the cloning of COR-L32 and COR-L65 cells ($10^5$ cells of each plated). The effect on PE of PBS and distilled water as solvents for the agarose was also examined. Colonies were counted 14 days after plating and the results are shown in Table V. For both cell samples, 'Sea Plaque' agarose produced higher PE than 'Sea Kem' agarose. There appears to be little difference in PE between PBS and distilled water when used as solvents for 'Sea Plaque' agarose and only a slight increase in clonogenicity of COR-L65 when PBS was used with 'Sea Kem' agarose. However, the combination of 'Sea Kem' agarose and PBS produced larger colonies than did the combination of 'Sea Kem' agarose and distilled water. There was no difference in the appearance of colonies produced between PBS and distilled water when used in combination with 'Sea Plaque' agarose.

In the Carney method, colonies rarely appeared before day 7 of incubation and because no replenishment of the medium is carried out in this method, experiments were generally scored before day 21. During this period of incubation the PE of most cell specimens increased (Table VI). Cultures of all specimens contained small clusters of $<50$ cells which were not scored. In the case of NCI-H69, these small clusters were scored separately from the usual colonies. The ratio of colonies:clusters was, on day 9 of incubation, 0:33; on day 12, 7:94; and on day 15, 17:72. It seems likely that by day 12, all cells destined to produce small clusters would have done so; some of these growing to consist of more than 50 cells during the interval from day 12 to day 15 and therefore scored as colonies not clusters.

**Table V** Effect of type of agarose and solvent for agarose on PE of 2 recent *in vitro* specimens in the Carney method

| | *PE*[a] | | | |
|---|---|---|---|---|
| | *'Sea Kem' agarose* | | *'Sea Plaque' agarose* | |
| *Specimen* | *Distilled water* | *PBS* | *Distilled water* | *PBS* |
| COR-L32 | 0.0 | 0.0 | $6.3 \times 10^{-4}$ | $4.8 \times 10^{-4}$ |
| COR-L65 | $9.3 \times 10^{-5}$ | $2.9 \times 10^{-4}$ | $3.3 \times 10^{-2}$ | $1.7 \times 10^{-2}$ |

[a]Colonies counted after 14 days in culture.

**Table VI** Effect of incubation time on PE in the Carney method

| Specimen | Experiment | Cells plated | PE at various time intervals (days) after plating 7–9 | 12–16 | 21–22 |
|---|---|---|---|---|---|
| NCI-H69 | | $2 \times 10^3$ | 0.0 | $3.3 \times 10^{-3}$[a] $8.7 \times 10^{-3}$[b] | ND |
| COR-L24 | I | $10^5$ | 0.0 | 0.0 | 0.0 |
| | II | $10^5$ | ND | $7.7 \times 10^{-4}$ | $1.7 \times 10^{-3}$ |
| COR-L32 | I | $10^5$ | $5.0 \times 10^{-5}$ | $2.4 \times 10^{-4}$ | $2.7 \times 10^{-4}$ |
| | II | $10^5$ | ND | $2.7 \times 10^{-4}$ | $6.7 \times 10^{-4}$ |
| COR-L47 | | $10^5$ | $6.2 \times 10^{-4}$ | $3.3 \times 10^{-3}$ | $2.3 \times 10^{-3}$ |
| COR-L51 | | $10^5$ | 0.0 | 0.0 | 0.0 |

[a]Counted on day 12; [b]Counted on day 15; ND, not done.

### Discussion

The PE was higher in the Courtenay method than in the Carney method for all lung cancer specimens tested. The difference between the 2 methods was most noticeable when comparing the PE of early culture specimens suggesting an advantage of the Courtenay method for specimens of very low PE. In both methods the PE of the established cell lines was generally much higher than those of the early culture specimens. This finding is not surprising as established cell cultures consist of a more homogeneous population of replicating cells and therefore may contain a higher proportion of clonogenic cells than do early cell cultures. Carney *et al.* (1980) have shown that the PE of various fresh specimens containing SCLC cells is ~1% of SCLC cells plated but only 0.02 to 0.25% of the total nucleated cells plated whilst the PE of 2 established cell lines was 1.0 to 5.6%. Interestingly Gazdar *et al.* (1980), using the Carney method, demonstrated that, of the established cell lines plated, the non-small cell lung cancer lines gave much higher PE (28 to 44%) than did the SCLC lines (0.03 to 5.2%). We have found the PE of our non-small cell and SCLC lines to be very similar using the Courtenay method.

It appears that normal bone marrow specimens can form colonies giving rise to similar PE in both methods. These results are somewhat surprising as although it is well established that mouse bone marrow can give rise to both macrophage and granulocyte colonies in agar, the formation of these colonies depends upon the presence of a colony stimulating activity (Bradley & Metcalf, 1966; Metcalf *et al.*, 1967; Bradley *et al.*, 1971). Using the Carney method, Carney *et al.* (1980) reported no colonies being formed from 7 bone marrow specimens histologically negative for SCLC. Although the PE of our normal bone marrow specimens in the Carney method was low, they still gave large colonies consisting of >50 cells that ranged in number from 2 (NMB 4) to 20 (NBM 3) for $10^5$ cells plated. The colonies formed from normal bone marrow were of 2 distinct types (1) diffuse groups of cells (not scored) and (2) tightly packed clusters of cells similar to colonies produced by SCLC specimens. Colonies of the latter type will make it difficult to select colonies from bone marrow specimens with SCLC infiltration if the specimen is cloned direct from the clinic.

There are several reasons which may explain why the Courtenay method produces higher PE than the Carney method. The two most critical factors appear to be the presence of rat RBC and low $O_2$ concentration. Bradley *et al.* (1971) first demonstrated that washed rat RBC improved the clonogenicity of mouse marrow cells and produced larger colonies. RBC lysed before use were found to be equally effective as whole cells in promoting colony growth suggesting the release of a growth factor. However, using human tumour xenografts, Courtenay & Mills (1978) found that RBC lysed before use were less effective than whole cells and that the time of lysis in culture is important. RBC from August rats were superior to RBC from other strains of rat because they lysed over a period of 5–7 days, a critical time for colony growth. Also, the PE of various cell types seems dependent on an optimal number of RBC (Bradley *et al.*, 1971; Courtenay & Mills, 1978; Courtenay, 1983). We found that there is a general requirement for rat RBC although the 2 non-small cell lines (COR-L23 & MOR) appear less dependent upon them than the NCI-H69 cell line. Indeed, NCI-H69 cells seem very sensitive to the concentration of RBC since the PE was found to be gradually reduced as the RBC were diluted from 1/8 to 1/64. In the absence of RBC, the PE of these cells was <50% of the PE at the lowest dilution.

Recently, Tveit *et al.* (1981*a*; 1981*b*) showed that when cloning melanoma xenografts 5% $O_2$ produces higher PE than does 20% $O_2$. In fact, many different tumour cell types appear to have better PE at $O_2$ concentrations of 5% or less (Richter *et al.*, 1972; Courtenay, 1976, 1985; Gupta & Krishan, 1982). Using the clonogenic assay of Hamburger & Salmon (1977), Gupta & Eberle (1984) demonstrated that although there was great variation between the PE of cells from the various human xenograft lines tested, the optimal concentration could be as low as 0.1%. These reports suggest that the most effective $O_2$ tension for improving the PE of cells in the clonogenic assay is in the range of 0.1–5%. Our data confirm these findings and show that gassing with $O_2$-free $N_2$ produces PE for several lung cancer cell types that are equal to or better than those obtained when 5% $O_2$ was used. Although there may have been some residual $O_2$ present after gassing, perhaps dissolved in the agar plug and/or plastic tube used in the assay method, it is unlikely to have been greater than 1% and therefore would not have influenced the results.

Courtenay (1983) recommends the use of HR cells to make up the number of cells plated to $10^4$. The function of HR cells is unknown but may be related to the production of growth factor(s) or the the consumption of $O_2$ which would help to maintain a low $O_2$ environment suitable for the formation of colonies. Although cells in culture derived recently from clinical specimens show an improvement in PE with HR cells, those from the well-established cell line, NCI-H69 do not. Also, in the absence of HR cells, most cell growth arising from recently derived cells is in the form of small clusters consisting of $<50$ cells. Thus it appears that cells which have been growing in culture for extended periods of time at elevated $O_2$ levels may be less dependent on a low $O_2$ concentration in the clonogenic assay than are cells more recently derived from tumours which may require an *in vitro* $O_2$ environment more similar to that found *in vivo*.

Factors influencing PE in both the Courtenay and Carney methods include incubation conditions and incubation time. It seems that an isotonic agar solution is advantageous for plating cells in the Courtenay method as agar prepared in PBS produced higher PE than agar prepared in distilled water. In the Carney method, there was no clear advantage of using PBS to prepare agarose. However, the use of 'Sea Plaque' agarose resulted in higher PE than was seen with 'Sea Kem' agarose regardless of the solvent used. Surprisingly, in both methods, PE were much higher in those cultures incubated in a walk-in warm room than in those incubated in the $CO_2$ incubator. If there was a degassing effect on cell growth in the warm room, one would expect the opposite results. Although there is no apparent reason for the PE to be higher in cultures in the warm room, such variation in cell growth has been known to occur. Buckmeir *et al.* (1984) have observed large variations in clonogenic cell growth between replicate cell plates placed in several different gassing incubators. These authors suggest that the different PE may be related to differences in water loss from the agar which was found to occur during incubation.

Prolonged incubation periods in the Courtenay method were necessary for specimens cloned direct from the patient and for 1 of 3 xenografts of early culture specimens (COR-L32). For all specimens except COR-L92 and COR-L93 which were diagnosed negative for tumour cells, there was a considerable increase in PE between 4 and 6 weeks. In one case (COR-L90) the clonogenicity was still increasing at 12 weeks. The Courtenay method has a particularly useful advantage over the Carney method as it is possible to replensih the medium and thus maintain slow-growing colonies over long periods of time. In fact the weekly replenishment of the medium over the usual 3 to 4 week period may be responsible for the generally higher PE seen routinely with the Courtenay method. It is apparent that, for the cells studied, an incubation period in excess of 9 days is required for colony formation in the Carney method. Even NCI-H69 plated at 5 times the number of cells used in the Courtenay method failed to produce colonies during the first week. Although 4 specimens showed a continual increase in PE up to 3 weeks, the cultures could not be maintained longer than 3 weeks because in the Carney method, the agarose is not replenished.

In conclusion we have shown that the Courtenay method provides higher PE than the Carney method for human SCLC cells including specimens direct from the patient, early cell cultures and established cell lines. The improved clonogenicity in the Courtenay method is due primarily to the presence of RBC and low $O_2$ tension although the replenishable nature of the assay allows for extremely long incubation periods often necessary for specimens cloned directly from the patient.

The authors wish to thank Professor Norman M. Bleehen for his continued support. Also, many thanks are due to Jane Hanson and Ann Coombes at the Radiobiology Unit, Velindre Hospital, Cardiff, for discussion of their unpublished work on the use of extended incubation times in the Courtenay method.

### References

BAILLIE-JOHNSON, H., TWENTYMAN, P.R., FOX, N.E. & 6 others. (1985). Establishment and characterization of cell lines from patients with lung cancer (predominantly small cell carcinoma). *Br. J. Cancer*. **52**.

BRADLEY, T.R. & METCALF, D. (1966). The growth of mouse bone marrow cells *in vitro*. *Aust. J. Exp. Biol. Med. Sci.*, **44**, 287.

BRADLEY, T.R., TELFER, P.A. & FRY, P. (1971). The effect of erythrocytes on mouse bone marrow colony development *in vitro*. *Blood*, **38**, 353.

BUCKMEIR, J.A., THOMSON, S.P., BREGMAN, M.D. & MEYSKENS, F.L. (1984). Marked variation of clonogenic growth between incubators and the loss of water during incubation. In: *Human Tumour Cloning*, Salmon & Trent (eds). Harcourt Brace Jovanovich: New York.

CARNEY, D.N., GAZDAR, A.F. & MINNA, J.D. (1980). Positive correlation between histological tumor involvement and generation of tumor cell colonies in agarose in specimens taken directly from patients with small-cell carcinoma of the lung. *Cancer Res.*, **40**, 1820.

COURTENAY, V.D. (1976). A soft agar colony assay for Lewis lung tumour and B16 melanoma taken directly from the mouse. *Br. J. Cancer*, **34**, 39.

COURTENAY, D. (1983). The Courtenay clonogenic assay. In *Human Tumour Drug Sensitivity Testing in vitro*, Dendy & Hill (eds), p. 103. Academic Press: London.

COURTENAY, V.D. (1985). Primary cultures in tumours. In *Mammalian Colony Regeneration Techniques*, Potten & Hendry (eds), Churchill Livingstone: Edinburgh.

COURTENAY, V.D. & MILLS, J. (1978). An *in vitro* colony assay for human tumours grown in immune-suppressed mice and treated *in vivo* with cytotoxic agents. *Br. J. Cancer*, **37**, 261.

GAZDAR, A.F., CARNEY, D.N., RUSSELL, E.K. & 5 others. (1980). Establishement of continuous, clonable cultures of small-cell carcinoma of the lung which have amine precursor uptake and carboxylation cell properties. *Cancer Res.*, **40**, 3502.

GUPTA, V. & KRISHAN, A. (1982). Effect of oxygen concentration on the growth and drug sensitivity of human melanoma cells in soft-agar clonogenic assay. *Cancer Res.*, **42**, 1005.

GUPTA, V. & EBERLE, R. (1984). Modulation of tumour cell colony growth in soft agar by oxygen and its mechanism. *Br. J. Cancer*, **49**, 587.

HAMBURGER, A.W. & SALMON, S.E. (1977). Primary bioassay of human tumor stem cells. *Science*, **197**, 461.

METCALF, D., BRADLEY, T.R. & ROBINSON, W. (1967). Analysis of colonies developing *in vitro* from mouse bone marrow cells stimulated by kidney feeder layers or leukemie serum. *J. Cell Physiol.*, **69**, 93.

RICHTER, A., SANFORD, K.K. & EVANS, V.J. (1972). Influence of oxygen and culture media on plating efficiency of some mammalian tissue cells. *J. Natl Cancer Inst.*, **49**, 1705.

TVEIT, K.M., FODSTAD, O. & PIHL, A. (1981*a*). Cultivation of human melanomas in soft agar. Factors influencing plating efficiency and chemosensitivity. *Int. J. Cancer*, **28**, 329.

TVEIT, K.M., ENDRESEN, L., RUGSTAD, H.E., FODSTAD, O. & PIHL, A. (1981*b*). Comparison of two soft-agar methods for assaying chemosensitivity of human tumours *in vitro*: Malignant melanomas. *Br. J. Cancer*, **44**, 539.